# Rational design of nonstoichiometric bioceramic scaffolds via digital light processing: tuning chemical composition and pore geometry evaluation

**DOI:** 10.1186/s13036-020-00252-3

**Published:** 2021-01-06

**Authors:** Yifan Li, Ronghuan Wu, Li Yu, Miaoda Shen, Xiaoquan Ding, Fengling Lu, Mengtao Liu, Xianyan Yang, Zhongru Gou, Sanzhong Xu

**Affiliations:** 1 Department of Orthopedics, the First Affiliated Hospital, Zhejiang University School of Medicine, #79 Qingchun Road, Hangzhou, Zhejiang Province 310003 P. R. China; 2 Operation Room, the First Affiliated Hospital, Zhejiang University School of Medicine, #79 Qingchun Road, Hangzhou, 310003 Zhejiang Province P. R. China; 3 Bio-nanomaterials and Regenerative Medicine Research Division, Zhejiang-California International Nanosystem Institute, Zhejiang University, #866 Yuhangtang Road, Hangzhou, Zhejiang Province 310058 P. R. China

**Keywords:** Pore geometry, Mechanical properties, Biodegradation, Nonstoichiometric wollastonite scaffolds, Ceramic stereolithography

## Abstract

Bioactive ceramics are promising candidates as 3D porous substrates for bone repair in bone regenerative medicine. However, they are often inefficient in clinical applications due to mismatching mechanical properties and compromised biological performances. Herein, the additional Sr dopant is hypothesized to readily adjust the mechanical and biodegradable properties of the dilute Mg-doped wollastonite bioceramic scaffolds with different pore geometries (cylindrical-, cubic-, gyroid-) by ceramic stereolithography. The results indicate that the compressive strength of Mg/Sr co-doped bioceramic scaffolds could be tuned simultaneously by the Sr dopant and pore geometry. The cylindrical-pore scaffolds exhibit strength decay with increasing Sr content, whereas the gyroid-pore scaffolds show increasing strength and Young’s modulus as the Sr concentration is increased from 0 to 5%. The ion release could also be adjusted by pore geometry in Tris buffer, and the high Sr content may trigger a faster scaffold bio-dissolution. These results demonstrate that the mechanical strengths of the bioceramic scaffolds can be controlled from the point at which their porous structures are designed. Moreover, scaffold bio-dissolution can be tuned by pore geometry and doping foreign ions. It is reasonable to consider the nonstoichiometric bioceramic scaffolds are promising for bone regeneration, especially when dealing with pathological bone defects.

## Introduction

The treatment of bone defects is currently a difficult orthopedic challenge and a large number of investigations have focused on it. A method to improve the osteogenesis of bone grafts and reductions in the amounts of autologous bone required is urgently required [1–3]. Therefore, the development of artificial, biodegradable bone-repair biomaterials, with mechanical properties that match the rate of new bone growth, is a valuable pursuit [4]. Additive manufacturing technology has promoted the research and development of bone regeneration [5, 6]. The most promising additive manufacturing techniques include three-dimensional (3D) printing, fused deposition modeling, selective laser sintering, stereolithography and so on [7–10]. Among the most important characteristics of the biomaterials created is their morphology, including pore shape, pore connectivity, and overall porosity [11, 12]. These factors play an important role in the process of bone regeneration. The three-dimensional (3D) porous structure should provide the necessary mechanical support for cells to proliferate and maintain their differential function and restore the initial shape of new bone [13]. Nowadays, Macroporous scaffolds with arbitrary internal structure and external morphology can be realized using the combination of computer-aided design (CAD) and layer-by-layer printing [14]. Meanwhile, the pore geometry and shape of the bioceramic scaffolds prepared by stereolithography can be accurately controlled to match the arbitrary and complex 3D anatomical shape of the bone defect [15, 16].

Wollastonite (CaSiO_3_; CSi) is a promising bioceramic candidate with good biocompatibility and biodegradability [17]. It has been demonstrated that CSi bioceramic can enhance bone regeneration and vascularization in vitro and in vivo by releasing calcium and silicon ions into the surrounding environment [18]. Moreover, its excellent apatite mineralization ability may contribute to its biological activity and promote osteogenesis in vivo [19]. However, pure CSi alone cannot be used as bone substitutes for load-bearing bone, due to the inherent shortcomings of high biodegradation rate and poor sintering property, which result in fast ion release, collapse of porous architecture, as well as poor mechanical support [20, 21]. Because of these limitations, researchers have been widely exploring new strategies to optimize the biodegradation rate and mechanical properties of CSi bioceramics [22]. One important strategy is the addition of therapeutic ions and bioactive substances to CSi bioceramics that optimize their degradation rates, improve their mechanical properties, and promote the repair of bone defects, especially in patients with basic pathological diseases such as osteoporosis [22–25].

Strontium (Sr) is the well-known, biologically active essential element in bone metabolism. It improves bone mineral density and anti-absorption in human bone tissue [26–28]. It was previously reported that Sr-doping in Ca-phosphate and Ca-silicate bioceramics may accelerate their bio-dissolution in vitro [23, 24]. Furthermore, when integrated into biomaterials, Sr can be administered directly into specific bone defects, which greatly reduces the necessary drug dosage and the risk of possible side effects [29, 30]. It is well-known that Sr can stimulate osteoblasts and inhibit osteoclasts in vitro, while magnesium (Mg) is important to bone metabolism, stimulates new bone formation, and increases bone cell adhesion [31–33]. Previous studies have shown that some kind of novel bioactive glasses and bioceramics containing Sr or Mg had an enhanced osteostimulating effect on new bone formation, thus indicating Sr^2+^ ions may improve the osteogenic activity of silicate ceramics [31, 34–36]. Therefore, it is reasonable to assume that Sr-doping of CSi-Mg may tune its bioactivity, biodegradability, and even mechanical stability.

In this study, we designed Mg/Sr co-doped wollastonite (CSi-Mg5Sr*x*) macroporous bioceramics with different concentrations of Sr (*x* = 0%, 2.5%, 5%, 10%), and we investigated the concentration-dependent changes in their biodegradable and mechanical properties in vitro. The porous scaffolds, with a variety of pore geometries, were fabricated using CAD and ceramic stereolithography [37]. The scaffolds were systematically characterized before and after immersion in biomimetic body solution media, such as SBF and Tris-HCl buffers. Their physiochemical and mechanical properties were evaluated based on pore geometry and Sr dopant content.

## Materials and methods

### Chemicals and materials

The reagent-grade inorganic salts were bought from Sinopharm Reagent Co., Shanghai, and used without further purification. The organic reagents, including 1,6-hexanediol diacrylate (HDDA; Alfa Aesar Co.), 6-trimethyl- benzoyldiphenylphosphine oxide (TBDPO; BASF (China) Co., LTD), and ethoxylated pentaerythritol tetraacrylate (PPTTA; DSM-AGI Co., LTD) were used directly without any pretreatment. Simulated body fluid (SBF; Kokubo’s recipe) was prepared in ultrapure water and buffered to a pH ~ 7.40 before 0.22-μm-membrane filtering [38]. Trishydroxymethylaminomethane (Tris) was used to prepare the 0.05 M Tris buffer (pH ~ 7.40).

### Synthesis of CSi-Mg5Sr*x* powders

The CSi-Mg5Sr*x* (Ca_100–5%-*x*%_Mg_5%_Sr_*x*%_SiO_3_; *x* = 0, 2.5, 5, 10) powders were synthesized using a wet-chemical precipitation method as previously described [39]. After calcinating at 850 °C for 2 h in a muffle furnace with a heating rate of 2 °C/min, the CSi-Mg5Sr*x* powders were ground with zirconia ball grounding media for 6 h. The particle size of the resulting powders was below 5 μm. The CSi-Mg5Sr*x* powder was verified by X-ray diffractometry (XRD; Rigaku D/max-rA) using CuKα radiation at 40 kV/40 mA. Data were collected between 10^o^ and 60^o^ to identify any crystallization of the powders. The contents of Ca, Mg, Si and Sr in the powders were measured using inductively coupled plasma-optical emission spectroscopy (ICP-OES: 710-ES, Varian; USA).

### 3D printing of porous scaffolds

The powder suspensions used for printing contained the following main components: curable monomers, photo initiators, dispersants and CSi-Mg5Sr*x* powders. HDDA and PPTTA were used as the monomers, with a mass ratio (mHDDA: mPPTTA) of 7:1. The volume loading rate of CSi-Mg5Sr*x* powder was approximately 60%. The powders and resins were mixed in a ball mill for 2 h. The wavelength of the stereolithography apparatus used for the curing ultraviolet (UV) light was 405 nm. The cured layer was placed directly on the glass sheet and irradiated with UV light from below. The intensity of decayed UV light above the cured layer was measured with a radiometer. The photosensitive area of the radiometer was an 8-mm-diameter circle. The cured layers were manufactured with a thickness between 50 and 60 μm.

**Scheme 1:**
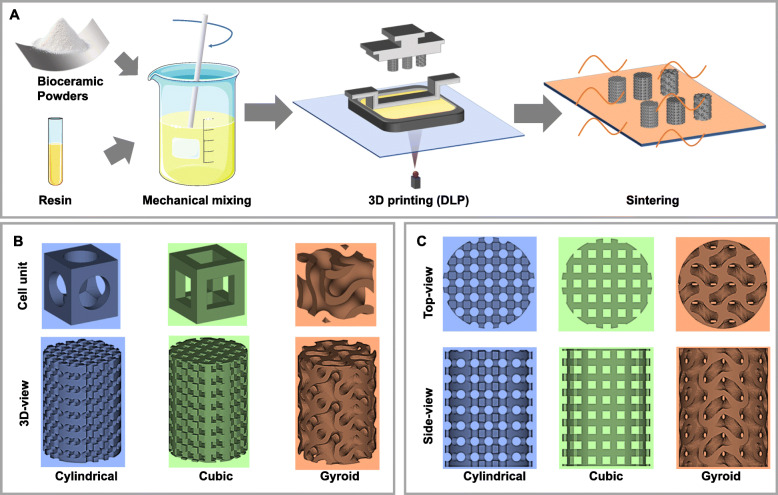
**a** Schematic diagram of the preparation process for manufacturing 3D porous bioceramic scaffolds with different pore geometries, including cylindrical, cubic, and gyroid pores designed by CAD. **b** Respective top and side views of the model images. **c** Designed 3D pore shape of the porous models and pore unit cells (Cylindrical, Cubic, Gyroid)

3D scaffolds with different shapes were designed using Magics software, including *Cylindrical*, *Cubic* and *Gyroid* structures (see Scheme 1). The cell structure of the Gyroid was created using MathMod software and characterized by Magics software, which of the Cylindrical and Cubic were only created and characterized by Magics software. The software of Avizo was used to calculate the average pore size and porosity of cell units to control the porosity and the average pore size of the three structures keeping respectively the same as ~ 58% and ~ 600 μm during the design stage for the porous scaffold. The corresponding porous scaffold was built by the “Structure” function in the software of Magics based on the cell unit created and authenticated by the above method. Therefore, they had similar sectional pore areas with approximate theoretical porosity. As shown in Scheme 1, the printed bodies were finally obtained by a process of stereolithography in the Autoceram Ceramic 3D Printer (Beijing Ten Dimensions Technology Co., Ltd., China) and based on the photo-polymerization of a mixture of resins and powders. Every layer was patterned and 3D structure was formed by stacking each layer of flats into the 3D pattern. The powder-resin slurry was poured into the printing tank, and applicable exposure parameters were selected to complete the bottom-up stereolithography process. After the printing process completed, the printed bodies were washed ultrasonically in deionized water, and then dried at 80 °C overnight. Finally, the porous bodies were sintered in a Muffle furnace at a target temperature of 1150 °C using similar heating schemes (the heating rate was 3 °C/min and 400 °C was maintained for 120 min) and held at the target temperature for 3 h. Afterward, they were allowed to cool naturally.

### Primary morphology and structure analysis

The external scaffold morphology and pore architecture were observed using a mobile camera (iPhone 10, Apple). The surface and fracture microstructures of the bioceramic scaffolds were investigated with scanning electron microscopy (SEM; JEM-6700F; Japan). Prior to examination, the macroporous samples were coated with a thin layer of gold. The linear shrinkage before and after sintering was determined by measuring the diameter and height of the cylindrical scaffolds before and after sintering using a digital sliding caliper.

### Porosity analysis

The suitability of design for the porous scaffolds was determined by the calculated porosity of modeled samples. The volume and pore strut of the porous architecture were taken from Magics 21 software and used to determine the theoretical porosity. Another zero-damage method was used to measure the real porosities of the sintered cylindrical porous scaffolds. The mass (*m*_*1*_) of the sintered porous scaffolds was measured on a scale, and then the diameter (*D*) and height (*H*) were measured by using a Vernier caliper. The real porosity of the scaffolds (*n* = 6) was calculated using the following equation:

$$ {\displaystyle \begin{array}{c}\mathrm{Real}\ \mathrm{porosity}=\left(1-V/{V}_s\right)\times 100\%\\ {}V={m}_s/{\rho}_0\\ {}{V}_s=\pi \times {\left(D/2\right)}^2\times H\end{array}} $$

where *V*, V_s_, *m*_*s*_, and *ρ*_*0*_ were the volume of the solid structure, the volume of the porous sample, the mass of the porous sample, and the density of low dosage of Mg-substituted wollastonite (*ρ*_*0*_ = 2.917 g/cm^3^).

### Mechanical testing

The compressive strengths (σ_c_) of the sintered bioceramic scaffolds (Ø 6 × 8 mm; *n* = 6) were tested using a universal testing machine (Instron 5566) and a 10 kN load cell at a crosshead speed of 0.5 mm min^− 1^. The young’s modulus (*E*_*c*_) was calculated from the slope of the linear elastic region of the stress-strain curves. The following formula was used for the calculation:

$$ {\displaystyle \begin{array}{c}{\sigma}_{\mathrm{c}}=F/A\\ {}{E}_c={\sigma}_{\mathrm{c}}/\varepsilon \end{array}} $$

where *F*, *A*, *E*, ε are the maximal load (N), the circle surface area perpendicular to the load direction (mm^2^), the compressive modulus (MPa), and the strain (mm/mm), respectively.

The specific compressive strength (σ_s_) and Young’s modulus (*E*_s_) of the sintered scaffolds based on the apparent density (*ρ*_*s*_), were calculated using the following equation:

$$ {\displaystyle \begin{array}{c}{\sigma}_s=F/{S}_s\\ {}{E}_{\mathrm{s}}={E}_c/{S}_s\end{array}} $$

The specific compressive strength (σ_*d*_) and Young’s modulus (*E*_*d*_) based on the minimum cross-sectional area (*S*_*s*_) perpendicular to the load direction, were calculated using the following equation:

$$ {\displaystyle \begin{array}{c}{\sigma}_d={\sigma}_{\mathrm{c}}/{\rho}_s\\ {}{E}_d={E}_c/{\rho}_s\\ {}{\rho}_s={m}_{\mathrm{s}}/{V}_{\mathrm{s}}\end{array}} $$

where *ρ*_s_ is calculated by the mass (*m*_s_) and apparent volume (V_s_) of the scaffolds, and *S*_*s*_ was measured by μCT analysis.

### In vitro bio-dissolution evaluation

The CSi-Mg5Sr10 bioceramic scaffolds, with three types of pore geometries, and the bioceramic scaffolds with increasing Sr contents (Ø6 × 8 mm; *n* = 9; *W*_*0*_) were respectively immersed in Tris buffer (0.05 M; pH 7.4; 37 °C) at a scaffold/buffer ratio of 1.0 g/50 ml for 8 weeks. The Tris buffer was replaced every 7 days. From 4 h to 14 days after soaking, the buffer (0.5 ml) was extracted for ICP-OES analysis, and fresh buffer (0.5 ml) was added. After immersion for 2–8 weeks, three scaffolds in each group were rinsed with absolute ethanol, dried, and weighed (*Wt*). The mass decay was calculated with the following formula: Mass Decrease = *W*_*t*_/*W*_*0*_ × 100%. The mechanical strength of the dried scaffolds was also determined with a universal testing machine.

### In vitro evaluation of surface re-mineralization

The porous scaffolds (Ø 6 × 8 mm) were immersed in 10 ml of simulated body fluid (SBF) with an inorganic ion content similar to that of human plasma (Na^+^, 142 mM; K^+^, 5 mM; Ca^2+^, 2.5 mM; Mg^2+^, 1.5 mM; SO_4_^2−^, 1 mM; HPO_4_^2−^, 1 mM; Cl^−^, 36 mM; HCO_3_^−^, 14 mM) [38]. During the immersion, which was done at 37 °C, we monitored the formation of biomimetic hydroxyapatite (HA) on the surface of the scaffolds. After soaking for 7 days, the scaffolds were washed with distilled water, observed with SEM, chemically analyzed with energy dispersive X-rays (EDX).

### Statistical analysis

The data were expressed as mean ± standard deviation (SD) and analyzed with one-way ANOVA. In all cases the results were considered statistically significant when the *p*-value was less than 0.05.

## Results

### Primary characterization of the bioceramic scaffolds

**Fig. 1:**
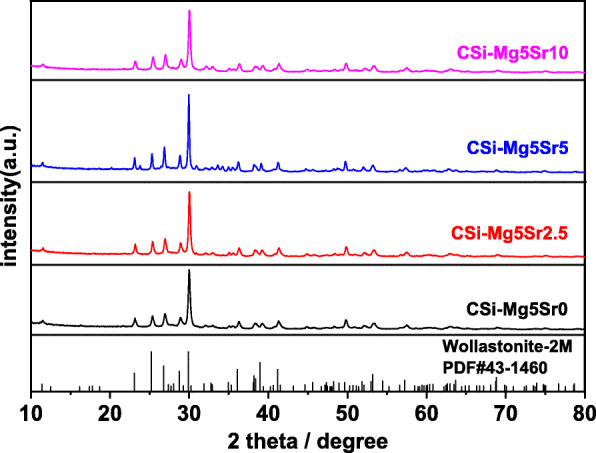
XRD patterns of the CSi-Mg5Sr*x* powders after calcination at 1150 °C

Figure 1 shows the XRD patterns of the CSi-Mg5Sr*x* bioceramic scaffolds. All diffraction peaks for all samples were identified as wollastonite-2 M phase (PDF #43–1460), suggesting that the Sr-substitution (up to 10%) led to no phase transformations of the CSi-Mg5. Moreover, the main diffraction peak shifts to higher or lower values due to the doping of Sr or Mg, indicating that the nonstoichiometric CSi was partly doped by the foreign ions. According to ICP analysis (Table 1), the real values of Mg or Sr substitution were similar to the theoretical ones. The Mg content in the CSi-Mg5Sr*x* (*x* = 0, 2.5, 5, 10) powders was 5.51–5.80%, and the Sr contents (0–9.5%) were increased with increasing Sr concentrations in the reaction solutions.

**Table 1:** ICP of different Sr and Mg co-doping CaSiO_3_ scaffolds

Powders	Ca (ppm)	Sr (ppm)	Mg (ppm)	Sr (mol%)	Mg (mol%)
CSi-Mg5Sr0	35.205	0	1.275	0	**5.51**
CSi-Mg5Sr2.5	39.995	2.920	1.500	3.05	**5.67**
CSi-Mg5Sr5	40.155	4.610	1.570	4.71	**5.80**
CSi-Mg5Sr10	37.840	8.805	1.560	9.52	**5.52**

### Structural evaluation of bioceramic scaffolds

The processing parameters and conditions used for the preparation of the porous samples were summarized in Table 2. The linear shrinkage of the sintered CSi-Mg5Sr*x* scaffolds displayed a steady increase from 15.5–16.3% for the CSi-Mg5Sr0 to 24.0–26.0% for the CSi-Mg5Sr5, but then decreased to 17.4–18.7% for the CSi-Mg5Sr10. It was worth noting, however, that there was no significant difference in shrinkage between the scaffolds with each of the three types of pore geometries. Additionally, as mentioned above, the real porosities were contrary to the fluctuations of linear shrinkage, even though the CAD-based porosities of the CSi-Mg5Sr*x* scaffolds were similar to each other during the model design.

**Table 2:** Structural parameters of the pores in the Mg5Sr*x* scaffolds

Pore geometry	Samples	Side-length/diameter of pore (μm)	Shrinkage (%)	CAD-porosity (%)	Real porosity (%)
Cylindrical	CSi-Mg5Sr0	D600	16.3 ± 4.2	54.3	53.8 ± 2.9
	CSi-Mg5Sr2.5		19.5 ± 0.7		54.1 ± 1.4
	CSi-Mg5Sr5		24.0 ± 0.8		50.3 ± 3.6
	CSi-Mg5Sr10		18.7 ± 6.0		53.1 ± 2.6
Cubic	CSi-Mg5Sr0	600	15.5 ± 1.7	55.2	53.5 ± 1.8
	CSi-Mg5Sr2.5		21.3 ± 1.0		49.0 ± 2.1
	CSi-Mg5Sr5		26.0 ± 2.9		46.7 ± 3.5
	CSi-Mg5Sr10		18.3 ± 1.1		57.9 ± 1.4
Gyroid	CSi-Mg5Sr0	D600	15.9 ± 0.9	57.2	55.9 ± 0.9
	CSi-Mg5Sr2.5		18.3 ± 1.2		59.2 ± 1.4
	CSi-Mg5Sr5		25.2 ± 0.3		54.1 ± 1.8
	CSi-Mg5Sr10		17.4 ± 1.4		55.6 ± 2.0

**Fig. 2:**
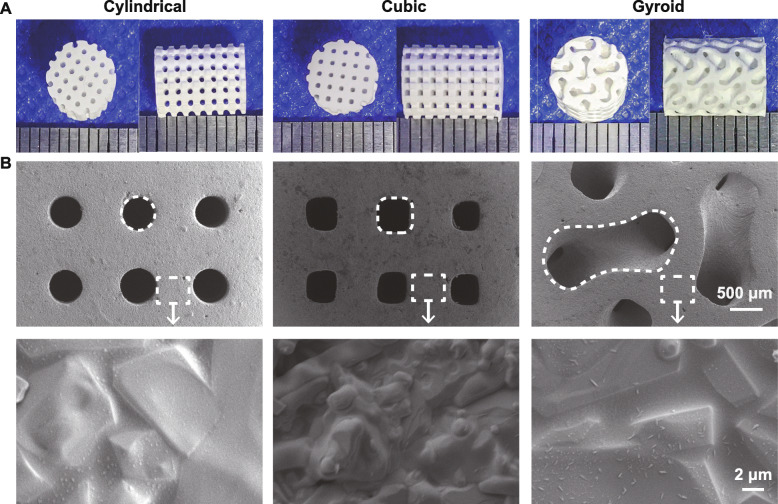
Outward appearance of the bioceramic scaffolds (**a**) and SEM images of the surface morphology and microstructures of the bioceramic scaffolds (**b**)

The outward appearance of scaffolds with precisely defined pore geometries after sintering was observed by digital camera (Fig. 2a). It was clear, from the top and side views, that the nearly cylindrical, cubical, or gyroid-curve pores were maintained. The scaffolds retained their porous architecture with no noticeable deformation of the total structure, except for some degree of shrinkage. Characterization of the sintered pore surface by SEM observation (Fig. 2b) clearly showed significant sintering densification and grain growth.

### Mechanical properties

**Fig. 3:**
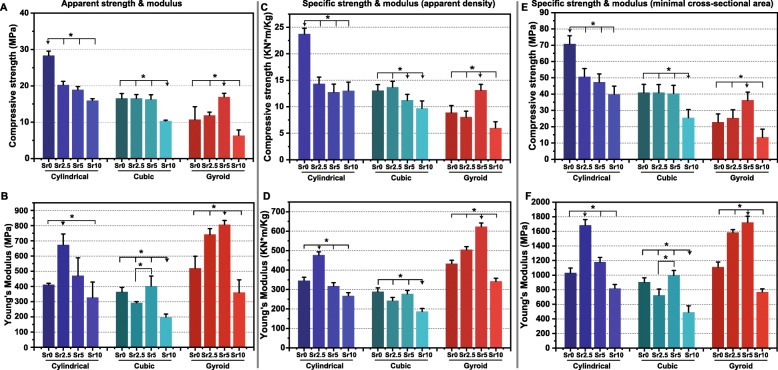
Apparent compressive strength (**a**) and Young’s modulus (**b**) of the CSi-Mg5Sr*x* scaffolds after sintering at 1150 °C. Specific compressive strength and Young’s modulus on apparent density (**c**, **d**) and minimal cross-sectional area (**e**, **f**) respectively of the bioceramic scaffolds after sintering. * *p* < 0.05

The apparent compressive strength and Young’s modulus of the bioceramic scaffolds were shown in Fig. 3. Overall, the cylindrical pore scaffolds had the highest compressive strength (≥16 MPa). The gyroid-pore CSi-Mg5Sr*x* scaffolds showed very low strength (≤12 MPa), except for the CSi-Mg5Sr5 variant. Interestingly, the cubic pore scaffolds maintained very stable compressive resistance (~ 16 MPa) until the Sr substitution was up to 10% (~ 10 MPa; Fig. 3a). On the other hand, it was interesting that the apparent Young’s modulus of the gyroid pore scaffolds was significantly higher than that of cubic pore scaffolds containing same Sr content (Fig. 3b). The specific strength and modulus of the scaffolds also showed similar results, which verified this point of view (Fig. 3c-f).

### Evaluation of degradation in vitro

**Fig. 4:**
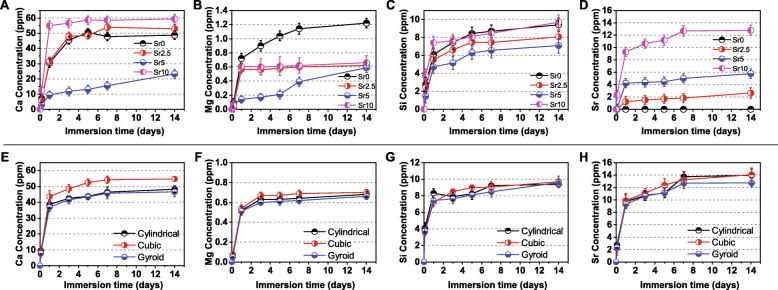
Bioactive ion release from the CSi-Mg5Sr*x* bioceramic scaffolds with different Sr concentrations (**a**–**d**) or different pore geometries (**e**–**h**) during immersion in Tris buffer for 14 days

The in vitro biodegradation of the bioceramic scaffolds with different pore geometries or Sr contents was monitored by calculating the ion concentration in Tris buffer. ICP analysis depicted the changes in ion concentration during the scaffold immersions (Fig. 4). As can be seen in Fig. 4a-d, the four types of inorganic ions (Ca, Mg, Si, Sr) released were directly mediated by the Sr dopant concentrations. The Ca and Mg release rates were promoted and inhibited, respectively, but the Si release rate was not regularly affected by Sr dopant concentrations. However, it was unexpected that the CSi-Mg5Sr5 scaffolds showed significantly slower ion release rates for Ca, Mg and Si in the early stages of immersion. Indeed, the higher the Sr content in the scaffolds, the higher the Sr ion concentration in the Tris buffer.

On the other hand, in Fig. 4e-h, the ions are released quickly within the first 24 h and then maintain a mild bio-dissolution rate in the buffer with the prolongation of immersion time. It was worth mentioning that, in total, the cubic pore scaffolds of CSi-Mg5Sr10 showed appreciable Ca, Mg, and Sr release in the early stages of immersion, but the gyroid pore scaffolds released ions slowly during the first 5 days.

**Fig. 5:**
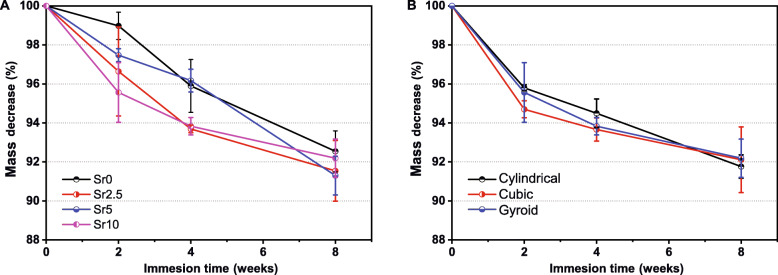
Mass decrease of the cylindrical CSi-Mg5Sr*x* (**a**) and Mg5Sr10 (**b**) bioceramic scaffolds during immersion in Tris buffer for 8 weeks

The mass decrease of the scaffolds mentioned above was measured during immersing in Tris buffer from 2 to 8 weeks, to simulate the microenvironment in the body (Fig. 5). The Sr-containing CSi-Mg5Sr*x* scaffolds showed more appreciable mass decrease (~ 2.5–4.3%) in comparison with the CSi-Mg5Sr0 (~ 1.0%) scaffolds in the initial 2 weeks (Fig. 5a), implying the bio-dissolution may be accelerated by Sr doping. The CSi-Mg5Sr5 scaffolds were sparingly soluble after 4 weeks but were undergoing rapid bio-dissolution at 8 weeks (~ 8.7% of mass loss). As expected, the CSi-Mg5Sr0 scaffolds were sparingly dissolvable in the whole immersion stage. Interestingly, the pore geometries of the CSi-Mg5Sr10 bioceramic scaffolds did not exhibit notable differences in mass decrease (Fig. 5b).

**Fig. 6:**
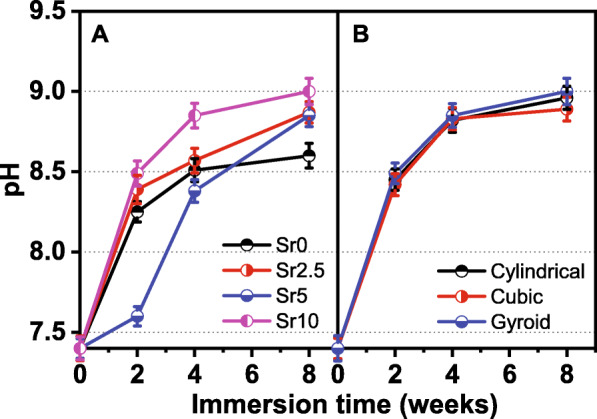
Changes in pH value in the Tris buffer during immersion of the CSi-Mg5Sr*x* bioceramic scaffolds with cylindrical pores (**a**) or composed of Mg5Sr10 (**b**)

**Fig. 7:**
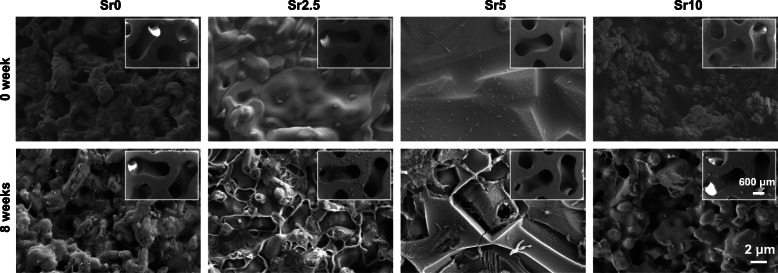
SEM images of the microstructural surface changes in the pore struts for the gyroid-pore CSi-Mg5Sr*x* scaffolds after immersion in Tris buffer for 8 weeks

The pH value in the Tris buffer increased gradually with the prolongation of immersion time, and Sr doping accelerated the process of pH increase. However, the different pore geometries had no significant effect on the change in pH value (Fig. 6). Moreover, the difference in the surface morphology of gyroid-pore scaffolds was characterized by SEM observations before and after the 8-week immersion. It can be seen from Fig. 7 that the pore wall became more noticeable rough and that the densification of the curved pores significantly decreased due to the bio-dissolution of the surface layer of the bioceramic struts.

**Fig. 8:**
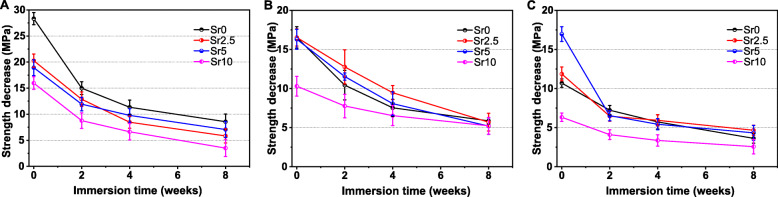
Strength decrease of the CSi-Mg5Sr*x* bioceramic scaffolds of cylindrical (**a**), cubic (**b**), and gyroid (**c**) pore geometries during the 8-week HCl-Tris buffer immersion

To further verify the strength decay during the immersion stage in Tris buffer, the compressive strength of bioceramic scaffolds was measured after immersing them for 2–8 weeks. As shown in Fig. 8, the compressive strength tended to decrease throughout the whole process. The cylindrical pore CSi-Mg5Sr*x* scaffolds maintained an appreciable compressive strength during the whole period of immersion, and the apparent strength was above 2.5 MPa after immersing for 8 weeks. Indeed, the CSi-Mg5Sr10 scaffolds exhibited structural stability after 8 weeks, though their compressive resistance was significantly lower than the other scaffolds in the whole immersion stage.

### Evaluation of surface re-mineralization in vitro

**Fig. 9:**
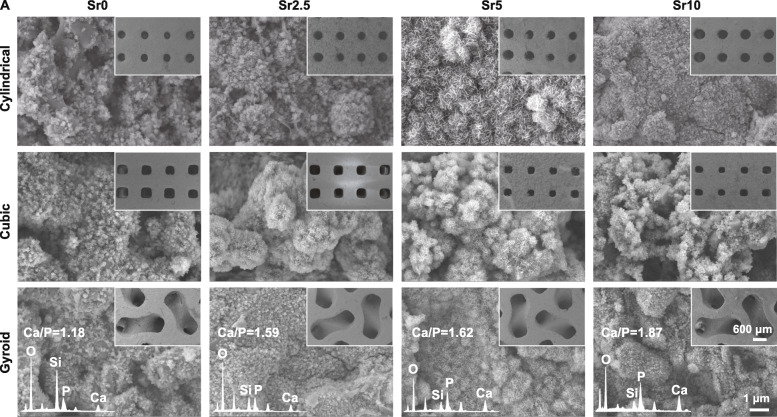
SEM observation of the CSi-Mg5Sr*x* scaffolds after soaking in SBF for 7 days. EDX spectra of the globular structures deposited on the surface of gyroid-pore scaffolds

The surface-inducing apatite re-mineralization bioactivity in vitro was characterized with SEM-EDX analysis. Figure 9 shows the SEM images of the scaffolds after immersing for 14 days in SBF. It can be seen that the pore geometries of the scaffolds were consistent before and after the immersion. Furthermore, it was observed that a dense new precipitate layer was coated onto the surface of the pore wall. According to the quantitative EDX analysis, there were significantly strong Ca and P peaks in the precipitate layer and the Ca/P ratio for the surface layer was ~ 1.18–1.87. The higher the Sr content in the bioceramic scaffolds, the higher the Ca/P ratio. This indicates that an apatite re-mineralization layer may readily deposit onto the pore walls.

## Discussion

It is well known that Mg ions can stimulate the proliferation and activity of osteogenic cells, inhibit the formation of osteoclasts, and promote angiogenesis [40, 41]. Some studies indicate that Mg could also enhance the sintering of glass-ceramics and bioceramics [42, 43]. According to our previous investigations, dilute Mg doped CSi ceramics, prepared by conventional pressureless sintering, show significantly enhanced mechanical strength and fracture toughness, but decreased biodegradation rates in vitro and in vivo [44–46]. Indeed, it is still a challenge to meet the osteogenic capacity and bone repair efficiency in critical-sized bone defects within an expected time window as long as the relationship between the pore structure, geometry, mechanical evolution, bioactivity, and biodegradation behavior of the ceramics is not elucidated. In this regard, we aim to develop Mg/Sr co-doped nonstoichiometric CSi scaffolds with appreciable mechanical strength and tunable biodegradation rates. The 3D printed CSi-Mg5Sr*x* scaffolds exhibit well-connected 3D porous architecture, appropriate pore size, and high porosity, which meet the essential conditions of the new generation of bone tissue-engineering scaffolds [4, 47].

It is reported that Sr-doped CSi ceramics have the ability to improve osteogenic differentiation in vitro and in vivo [18], and that Sr and Si have synergistic effects on osteoblasts, osteoclasts, and angiogenesis [35]. Previous studies have shown that Sr can also play a rapid role in promoting bone regeneration in the presence of Ca and Mg [48]. However, the ion radii of Sr and Ca are not the same [49]. Thus, the addition of Sr into calcium-based bioceramics will affect the stability of the lattice, which will impact the phase-transition temperature, grain geometry, and densification during sintering, which will ultimately affect their mechanical strength [49–51]. The changes to the stability of the lattice will also affect the degradation and ion release kinetics, which may affect the biological activity of the materials [21, 49, 52]. Furthermore, the anti-osteoporotic effect of Sr ions strongly depends on ion dosage, so it is of great significance to study the incorporation of different amounts of Sr ions into CSi-Mg bioceramic scaffolds [40].

In this study, we verified the mechanical strength, biodegradability, and other in vitro properties of CSi-Mg5Sr*x* scaffolds with different pore geometries, to determine the appropriate Sr-substituting concentration, and find a compromise between mechanical properties and biodegradation (including ion release-related bioactivity). XRD and ICP analysis confirmed that the Sr-substituted bioceramics, with corresponding content levels, were successfully prepared, and that all of them were nonstoichiometric CSi, free of second phases. According to previous studies on Ca-silicate systems, Sr ≤ 15% substitution is necessary to minimize the formation of unwanted second phases that may affect physical, chemical, and biological properties [18, 21, 50]. Because the radius of Sr ions is different from that of Ca ions [49], the lattice stability in the CSi bioceramics will be affected. Theoretically, the porous scaffolds containing different Sr contents need different sintering temperatures to obtain the best densification. In fact, the linear shrinkage and real porosity of the bioceramic scaffolds vary with the increase of Sr content, which may be related to Sr doping or sintering temperature in the present study [53]. Accordingly, the compactness of porous bioceramic scaffolds is possibly reduced due to high Sr dopant concentrations, and the compressive strength of porous bioceramic scaffolds decreases when Sr substitution is over 5% [54]. As a result, the compressive strength of CSi-Mg5Sr10 scaffolds is significantly lower than that of the CSi-Mg5Sr0 counterparts, which may be the result of under-sintering. In contrast, we found that the compressive strength of curved pore scaffolds is relatively low under the condition of similar porosity. This just indicates that the curvature and complexity of the pore wall/support have an important influence on the mechanical strength of the bioceramic scaffold. It is worth noting that the Young’s modulus of the gyroid pore scaffold is the highest among the three types of scaffolds, which implies that it has the best anti-deformation ability. Generally speaking, the mechanical strength of porous implants must be within the range of bone mechanical properties to avoid the stress shielding effect or the rapid collapse of the structure [55]. Some CSi-Mg5Sr*x* scaffolds readily meet the requirements necessary to integrate into cancellous bone defect, and thus, are promising in the clinical applications.

As for the biodegradability of bioceramic scaffolds, the results of immersion in Tris buffer were evaluated. First, we confirm that the Sr doping can accelerate the degradation of the bioceramic scaffolds and that this degradation is proportional to the Sr content level. Interestingly, the co-doping of Sr ions may accelerate the release rate of Ca and Si ions but reduce the release rate of Mg ions while maintaining the degradation rate of the bioceramics, thus changing the pH value of the solution. There is a certain correlation between the Sr release rate and its concentration in scaffolds. In addition, the biodegradation rate of gyroid pore scaffolds is the fastest among the three groups of scaffolds with different pore geometries, which is mainly attributed to its higher specific surface area. This tunable biodegradation rate of the bioceramic scaffolds is thought to be conducive to early osteoblast activity. New bone growth spurred by this bioactivity provides additional benefits, including structural stability and mechanical reliability for bone defect repair [54]. On the other hand, apatite can grow on the pore wall of scaffolds in SBF, which implies that the scaffolds can readily integrate with host bone tissue and produce low inflammatory reactions [38]. As we expect, the biomimetic apatite-like Ca-phosphate layer may deposit on the pore wall surface of all scaffolds, demonstrating its ability to induce bone mineral-like inorganic compound growth. Indeed, it may be speculated that the curve surface in the gyroid pore scaffolds is potentially favorable for cell adhesion and growth. Therefore, we believe that the ceramic stereolithography technique is very promising since optimization of the pore geometry is under investigation and promises to yield more intriguing properties.

## Conclusion

In summary, four groups of CSi-Mg5Sr*x* bioceramic powders with different Sr contents were successfully prepared. They were used in ceramic stereolithography to fabricate porous scaffolds with three types of pore geometries. A suitable concentration of Sr dopant can be applied to adjust the physicochemical and mechanical properties of CSiMg5 bioceramic. Sr can gently promote biodegradation in vitro and has appreciable bioactivity, though its use leads to a minor decline in compressive strength. The studies herein show that the mechanical strength and Young’s modulus of the bioceramic scaffolds are directly related to the curved surface-based (gyroid) and strut-based (cylindrical, cubic) pore topologies, and that the gyroid pore scaffolds have higher elastic moduli. Future work may concentrate on the early-stage bone repair efficacy of these foreign ion co-doped bioceramic scaffolds with different pore architectures, which are readily fabricated via stereolithography. Additionally, we recommend the use of this AM technique to develop a variety of high-strength bioceramic scaffolds with precisely tuned pore structures. Their use will lead to more efficient treatments of load-bearing large segmental bone defects in the nearby future, especially from the point of view of osteoporotic or pathological fracture conditions.

## Data Availability

The datasets used and/or analyzed during the current study are available from the corresponding author on reasonable request.

## Abbreviations

CSi: CaSiO_3_, Wollastonite
Sr: Strontium
Mg: Magnesium
Ca: Calcium
SBF: Simulated Body Fluid
3D: Three-dimensional
CAD: Computer-aided design
